# Effects of variable-intensity interval training and moderate-intensity continuous aerobic exercise on executive function and underlying neural mechanisms in male college students

**DOI:** 10.3389/fpsyg.2025.1643698

**Published:** 2025-11-10

**Authors:** Haoqin Si, Chen Yan, Tong Zhu, Mengqi Liu, Ziyang Yang, Niyuan Hu, Ying Qin, Liang Mu

**Affiliations:** Graduate School, Harbin Sport University, Harbin, China

**Keywords:** interval training, aerobic exercise, executive function, neural mechanisms, male college students

## Abstract

This study involved 51 male college students who were recruited and randomly assigned to one of three groups: a variable intensity interval training (VIIT) group, a moderate-intensity continuous training (MICT) group, and a control group (*n* = 17 each). Interventions are carried out for 6 weeks and three times a week, and the total duration of each test is about 53–61 min. Participants in the two intervention groups engaged in cycling exercises of different intensities, the entire experiment was conducted in a climate-controlled laboratory environment maintained at a constant temperature of 23 ± 1 °C. and the executive function and prefrontal hemodynamic responses of the participants were assessed before and after the intervention. Outcome measures included accuracy (ACC), reaction time (RT), and changes in oxyhemoglobin concentration (ΔHbO_2_). Postintervention results indicated that both the VIIT and MICT groups had significantly greater ACC in inhibitory control tasks compared with preintervention values and that of the control group (*P* < 0.05). RTs were significantly reduced in both exercise groups compared with their baseline values and that of the posttest control group (*P* < 0.05), whereas no significant differences were observed in the control group (*P* > 0.05). Hemodynamic data revealed significantly increased ΔHbO_2_ after VIIT in channels 41, 42, and 44 (dorsolateral prefrontal cortex), channels 26 and 27 (frontopolar area), and channel 4 (ventrolateral prefrontal cortex) (*P* < 0.05). In the MICT group, channels 42, 27, and 4 also presented significant increases in ΔHbO_2_ (*P* < 0.05). With respect to updating functions, RT significantly decreased postintervention in both exercise groups (*P* < 0.05), with the VIIT group showing shorter RT than the control group (*P* < 0.05) and the MICT group displaying greater ACC than controls (*P* < 0.05). VIIT elicited significant increases in ΔHbO_2_ in channels 40 and 43 (frontopolar area) and channel 41 (*P* < 0.05), whereas MICT resulted in significant activation only in channel 40 (*P* < 0.05). For the task-switching function, both the VIIT and MICT groups demonstrated significantly reduced RTs after training (*P* < 0.05), with no significant change in the control group. In the VIIT group, channels 42, 44, and 4 presented significant increases in ΔHbO_2_ (*P* < 0.05). In conclusion, both VIIT and MICT effectively improved executive function performance in male college students, VIIT exhibited a more pronounced increase in prefrontal ΔHbO_2_, with activation primarily localized to the frontopolar, dorsolateral, and ventrolateral regions, whereas MICT also demonstrated noticeable activation in several brain regions.

## 1 Introduction

In recent years, exercise intervention has emerged as an increasingly prominent strategy for improving executive function (Hillman et al., 2008; Chang et al., 2017; Fan et al., 2021). Executive function, a core component of higher-order cognitive processes, is generally categorized into three major domains: inhibitory control (also referred to as inhibition), working memory (also known as updating), and cognitive flexibility (also termed task switching) (Diamond, 2013; Perner and Lang, 1999). The positive effects of exercise on cognitive function can be explained through multiple physiological mechanisms. From a hemodynamic perspective, aerobic training significantly enhances cerebral blood flow, particularly in the prefrontal cortex, thereby optimizing the energy supply required for cognitive task performance. On the neural level, exercise activates functional networks within the prefrontal cortex, promoting synaptic plasticity and improving neural connectivity (Miyake et al., 2000). In addition, exercise-induced release of neurotrophic factors—such as brain-derived neurotrophic factor (BDNF)—provides a molecular foundation for enhanced neuroplasticity and cognitive improvements (Yu et al., 2025).

Regarding the relationship between exercise intensity and executive function, evidence from Wang and colleagues suggests an inverted U-shaped relationship, where overly intense exercise may lead to temporary suppression of prefrontal activity (Wang et al., 2013; Coles and Tomporowski, 2008; Zheng et al., 2021; Wu et al., 2019). A meta-analysis by Mcmorris and colleagues further confirmed that moderate-intensity aerobic exercise promotes improvements in working memory (McMorris et al., 2011). These findings collectively suggest that the cognitive benefits of exercise are both intensity-dependent and component specific. VIIT elicits stronger sympathetic activation, greater cerebral blood flow fluctuations, and a more pronounced excess post-exercise oxygen consumption (EPOC) effect through high-intensity intermittent stimuli, potentially leading to more effective increases in prefrontal oxygenation and neuroplasticity. In contrast, MICT promotes the release of BDNF and cerebrovascular adaptation via continuous, stable aerobic metabolic stimulation. This study innovatively employed fNIRS technology and controlled the total energy expenditure across both exercise modes to investigate the differential effects of the two protocols on executive function performance and prefrontal hemodynamics in male college students. In this study, we employed the American College of Sports Medicine (ACSM) classification to quantify aerobic intensity based on percentage of maximum heart rate; moderate intensity and vigorous intensity exercise were classified respectively as 60–69% and 70–79% of the age-predicted maximum heart rate (Audiffren et al., 2009; Hanssen et al., 2018). The age-predicted maximum heart rate was calculated using the standard formula: 220 minus age. Future research should explore the dose-response relationship between exercise intensity and different components of executive function using frameworks such as dual-mode theory and prefrontal cortex activation models.

As the most evolutionarily advanced brain region, the prefrontal cortex (PFC) plays a central role in regulating higher-order cognitive functions (Pruessner et al., 2010). This anatomically complex structure comprises several functionally specialized subregions, including the dorsolateral prefrontal cortex (DLPFC), orbitofrontal cortex (OFC), ventrolateral prefrontal cortex (VLPFC), and frontopolar area (FPA). Functionally, the frontopolar area—known for its highest neuronal density and largest volume within the PFC—plays a critical role in complex cognition (Semendeferi et al., 2001; Dong et al., 2019). The DLPFC is responsible for maintaining and manipulating information in working memory and serves as a core node within the cognitive control network. Notably, the left and right hemispheres exhibit lateralization: the left DLPFC is involved primarily in semantic processing and retention (McReynolds et al., 2014), whereas the right DLPFC specializes in spatial information processing (Walker and Van Der Helm, 2009). The VLPFC plays a key role in memory formation and consolidation and works in concert with the DLPFC to regulate inhibition, task switching, and conflict monitoring (Walker and Van Der Helm, 2009). Pioneering research by David et al. has laid important groundwork for integrated exercise-cognition interventions (Clark et al., 2021), suggesting that enhanced PFC activity may underlie the improved executive control observed following such interventions. In this context, the current study employs functional near-infrared spectroscopy (fNIRS) to continuously monitor prefrontal cortex activity in response to aerobic exercise at varying intensities. This approach aims to elucidate the dose-response relationship between exercise intensity and executive function improvements while also examining the influence of inter-individual variability on intervention outcomes.

## 2 Materials and methods

### 2.1 Participants

A total of 51 male undergraduate students were recruited based on predefined inclusion and exclusion criteria. Participants were assigned to three groups using a completely randomized design: the variable intensity interval training (VIIT) group (*n* = 17), the moderate-intensity continuous training (MICT) group (*n* = 17), and the control group (*n* = 17). No significant differences were observed among the three groups in age, height, weight, or body mass index (BMI) (*P* > 0.05). The study was approved by the Ethics Committee of Harbin Sport University, and all participants provided written informed consent prior to participation. Basic demographic information is presented in Table 1.

**Table 1:** Basic characteristics of subjects (Mean ± SD).

**Group**	**VIIT (*n* = 17)**	**MICT (*n* = 17)**	**Control group (*n* = 17)**	***F*-value**	***P*-value**
Age (years)	20.71 ± 1.36	21.76 ± 1.79	20.71 ± 1.36	2.769	0.073
Height (m)	73.59 ± 5.94	76.82 ± 5.56	73.2 ± 6.35	1.471	0.240
Weight (kg)	1.76 ± 0.04	1.75 ± 0.05	1.77 ± 0.04	1.894	0.162
BMI (kg/m^2^)	23.71 ± 1.8	23.98 ± 1.71	23.34 ± 1.8	0.559	0.576

The data are presented as the mean ± standard deviation (Mean ± SD). VIIT, variable intensity interval training; MICT, moderate-intensity continuous training.

Inclusion criteria: male undergraduate students, aged 18–25 years, in good general health, with normal color vision and normal limb mobility sufficient to perform the required exercise tasks.

Exclusion criteria: any cardiovascular or metabolic disease, history of brain trauma or psychiatric disorders, severe respiratory or musculoskeletal conditions that would impede physical activity; consumption of alcohol- or caffeine-containing beverages within 48 h before the experiment; current or past smoking or nicotine dependence (which may affect cerebral hemodynamics); or poor fNIRS signal quality.

### 2.2 Experimental design

#### 2.2.1 Experimental protocol

A 3 (group: VIIT, MICT, control) × 2 (time: pretest, posttest) mixed factorial design was employed. The group (VIIT, MICT, control) served as the between-subjects factor, whereas the time (preintervention vs. postintervention) served as the within-subjects factor. The independent variables were exercise intensity and intervention duration, and the dependent variables included the change in oxyhemoglobin concentration (ΔHbO_2_), task accuracy (ACC), and reaction time (RT). Participants underwent a 6-week exercise intervention, with three sessions per week, each lasting ~33–36 min. The total duration of the executive function assessment was approximately 20–25 min, and the tasks were administered in a fixed order: the Stroop task, followed by the 2-back task, and then the More-odd shifting task.

During the experiment, after participants were properly fitted with a fNIRS device and heart rate monitor, the researchers calculated each individual's target heart rate range based on age, resting heart rate, and intended exercise intensity using a standardized formula. Participants in the VIIT and MICT groups performed cycling exercises at different intensities using a power-controlled ergometer, whereas those in the control group remained sedentary under normal conditions without any exercise intervention. fNIRS signals and heart rate data were continuously monitored throughout the experiment. Thirty seconds before the end of each exercise and recovery phase, participants' subjective ratings of perceived exertion (RPE) and real-time heart rate values were recorded. Participants were required to maintain a pedaling cadence of 60–70 rpm. RPE data were used solely for exercise-intensity monitoring and were not included in the statistical analyses. All participants familiarized themselves with the cognitive tasks before formal testing and achieved ≥85% accuracy. All sessions were conducted in a climate-controlled laboratory environment maintained at a constant temperature of 23 ± 1 °C. All experiments were conducted between 8:00 a.m. and 11:00 p.m. to control for the potential influence of circadian rhythms on cognitive performance.

#### 2.2.2 Exercise intervention protocol

Participants in the experimental groups engaged in two distinct exercise modes: VIIT and MICT. The control group watched a video for 30 min. A 6-week intervention was conducted, with three sessions per week, each lasting approximately 33–36 min. The energy expenditure during cycling was estimated using the metabolic equation provided in th*e ACSM's Guidelines for Exercise Testing and Prescription* (Thompson et al., 2013):


$$\begin{array}{c} {\text{VO}}_{2}=7.0+(10.8\times watt)/body\;mass, \end{array}$$ (1)

where VO_2_ is expressed in mL·kg^−1^·min^−1^. The workload (watts) on the ergometer served as the primary parameter to define exercise intensity. On the basis of this equation, the appropriate workloads were set as follows: 50 watts for both the warm-up and MICT phases, 100 watts for the VIIT high-intensity phase, and 30 watts for the VIIT recovery phase. The average body mass of the 51 participants was 75.01 kg.

To ensure energy equivalency, the total oxygen consumption for each intervention was first calculated based on the respective intensities and durations. Then, by applying the principle of energy conservation, the MICT session duration was adjusted to match the total energy output of the VIIT session. As a result, the calculated durations were as follows: MICT = 36.3 min and VIIT = 33 min (calculated as 499.04 mL/13.75 mL = 36.3 min). Details are provided in Table 2.

**Table 2:** Schematic diagram of phases, time, and intensity of VIIT and MICT.

**Group**	**Warm-up stage**	**Exercise stage**	**Recovery stage**	**Exercise stage**	**Recovery stage**	**Exercise stage**	**Recovery stage**
VIIT	40–60% HRR	75–85% HRR	30–60% HRR	75–85% HRR	30–60% HRR	75–85% HRR	30–60% HRR
	50 W	100 W	30 W	100 W	30 W	100 W	30 W
Time/min	10 min	4 min	3 min	4 min	3 min	4 min	5 min
Group	Warm-up stage	Exercise stage					
MICT	40–60% HRR	40–60% HRR	40–60% HRR	40–60% HRR	40–60% HRR	40–60% HRR	40–60% HRR
	50 W	50 W	50 W	50 W	50 W	50 W	50 W
Time/min	13 min	4 min	3 min	4 min	3 min	4 min	5 min

VIIT, variable intensity interval training; MICT, moderate-intensity continuous training; HRR, heart rate reserve.

Resistance levels were monitored and adjusted in real time by the research staff. During the warm-up, a progressive load protocol was used, starting at 15 watts and increasing by 5 watts every 30 s until reaching 50 watts. In the VIIT phase, resistance increased by 5 watts every 10 s from the 50-watt warm-up baseline until reaching a target of 100 watts, which was then maintained. The VIIT recovery phase used a rapid reduction protocol, in which the resistance was decreased from 100 to 30 watts with no fixed time limit. The MICT phase maintained a constant resistance of 50 watts. Pilot testing indicated that most participants reached their target heart rates within 1–2 min during the VIIT phase, whereas target heart rates were typically achieved by the end of the warm-up in the MICT group.

#### 2.2.3 Executive function tasks

**Figure 1:**
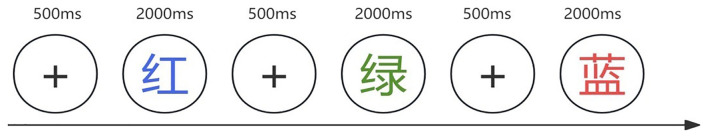
Stroop Task. The words “red,” “green,” “yellow,” and “blue” are presented in either congruent or incongruent colors. Each trial begins with a 500-ms white fixation point, followed by a 2,000-ms color-word stimulus.

**Figure 2:**
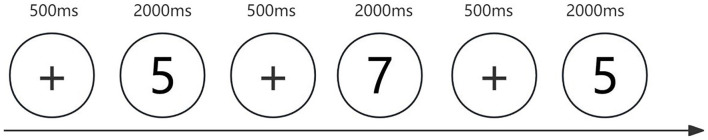
2-back Task. The stimuli are Arabic numerals from 1 to 9. Participants are required to match the current number with the one presented two trials before. Each trial begins with a 500-ms white fixation point, followed by a 2,000-ms numerical stimulus.

**Figure 3:**
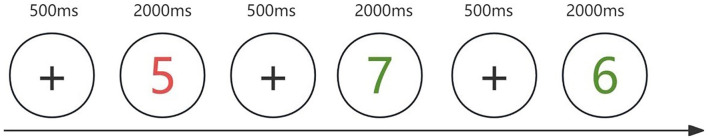
More-odd shifting. When the number is red, participants make a size judgment (compared to 5); when the number is green, they make an odd-even judgment. Each trial begins with a 500-ms white fixation point, followed by a 2,000-ms numerical stimulus.

Three computerized tasks were used to assess different components of executive function, all of which were programmed using E-Prime 3.0 software. Inhibitory control was measured using the Stroop color-word task. Stimuli consisted of Chinese color words (“red”, “green”, “yellow”, and “blue”) presented in either congruent or incongruent font colors. Participants were instructed to ignore the meaning of the word and respond based on the font color using specific keys (D for red, F for green, J for yellow, and K for blue) (Figure 1). Updating (working memory) was assessed using a 2-back task. Participants viewed a stream of digits (1–9) and were asked to determine whether the current number matched the one presented two trials earlier. They responded with the “F” key for matches and the “J” key for non-matches (Figure 2). Cognitive flexibility was assessed using the more-odd shifting task. Digits from 1 to 9 (excluding 5) appeared either red or green. If the number was red, participants judged whether it was greater or less than 5 (*F* for >5, J for < 5). If the number was green, they judged whether the number was odd or even (*F* for odd, J for even) (Figure 3).

Each task began with a 500-ms white fixation point, followed by a 2,000-ms stimulus presentation. The trial proceeded automatically upon participant response. Each task included a practice session (16 trials; repeated if accuracy < 85%; all participants met the accuracy criterion after 1–3 practice blocks; none were excluded for failing to reach the threshold.) and a formal testing phase comprising three blocks of 36 trials each, with 30-s intervals between blocks. Participants were instructed to respond as quickly and accurately as possible. The primary outcome variables were RT—measured from stimulus onset to key press—and ACC, defined as the proportion of correct responses.

### 2.3 Testing

#### 2.3.1 Prefrontal hemodynamic data acquisition

A portable fNIRS system (NirSmart, produced by HuiChuang Medical Equipment Co., Ltd., China) was used to continuously monitor hemodynamic responses in the prefrontal cortex of participants. Spatial registration of each channel to its corresponding brain region was performed using the Montreal Neurological Institute (MNI) coordinate system, which is based on a standard brain template. The corresponding Brodmann areas and their spatial coverage for each channel are presented in Table 3.

**Table 3:** Frontal lobe channel partitioning.

**Brain area**	**Passageway**
DLPFC	22, 24, 32, 38, 39, 41, 42, 44, 45, 47
FPA	6,8,11,21,23,25,26,27,28,40,43
VLPFC	4, 5, 19, 29, 31, 37, 46
OFC	7, 9, 10

DLPFC, dorsolateral prefrontal cortex; FPA, frontopolar area; VLPFC, ventrolateral prefrontal cortex; OFC, orbitofrontal cortex.

#### 2.3.2 Data processing

The raw behavioral data from the three cognitive tasks were integrated using the system's “merge” function module. The primary behavioral outcomes were ACC, calculated as the proportion of correct responses to total trials, and RT, defined as the interval between stimulus presentation (Chinese characters or digits) and the participant's key response.

fNIRS data were preprocessed using NirSpark software (version 1.0; HuiChuang Medical, China). First, a wavelet motion artifact correction algorithm (WMAC) was applied to remove signal distortions caused by head or body movements. Then, a zero-phase digital bandpass filter (0.01–0.2 Hz) was used to eliminate high-frequency physiological noise (e.g., heart rate variability, 0.8–2.5 Hz) and low-frequency drift (e.g., Mayer waves, 0.04–0.15 Hz) (Hu et al., 2019). Finally, dual-wavelength optical signals at 730 nm and 850 nm were converted into relative concentration changes of oxygenated hemoglobin (oxy-Hb), deoxygenated hemoglobin, and total hemoglobin using the modified Beer–Lambert law (MBLL). Given the greater sensitivity and specificity of oxy-Hb to neural activity (Hoshi et al., 2001), this study focused on changes in oxy-Hb concentration as the primary indicator of cortical activation.

### 2.4 Statistical analysis

All data are presented as the means ± standard deviation (Mean ± SD). Statistical analyses were performed using SPSS version 27.0. Data normality was assessed using the Shapiro–Wilk test, and the significance level was defined as a difference less than α = 0.05.

To compare baseline demographic characteristics (age, height, weight, and BMI) across groups, one-way analysis of variance (ANOVA) was employed. For normally distributed data, two-way repeated-measures ANOVA was used to evaluate the effects of time (pretest vs. posttest) and group (VIIT, MICT, control). For data that did not meet the assumption of normality, a non-parametric two-way ANOVA was applied. Effect sizes were reported using partial eta squared (ηp^2^), with values interpreted as follows: ηp^2^ < 0.06: small effect; 0.06 ≤ ηp^2^ < 0.14: medium effect; ηp^2^ ≥ 0.14: large effect.

### 2.5 Research team

All testing and training sessions were conducted by graduate students who had completed standardized training; a laboratory instructor was present for equipment calibration and on-site guidance, while the principal investigator oversaw the entire experimental process, data integrity, and ethical compliance throughout the study.

## 3 Results

### 3.1 Behavioral results of inhibitory control during the stroop task

As shown in Table 4, analysis of variance revealed no significant differences in baseline ACC among the three groups (*F*_(2, 45)_ = 3.066, *P* = 0.056, ηp^2^ = 0.120), indicating that participants were at a comparable baseline level. Postintervention, ACC in both the VIIT and MICT groups significantly increased compared with preintervention values (*P* < 0.001), whereas no significant change was observed in the control group (*P* = 0.921). A significant group effect was observed in the posttest comparison (*F*_(2, 45)_ = 145.958, *P* < 0.001, ηp^2^ = 0.866).

**Table 4:** Comparative analysis of stroop accuracy rates for three groups.

**Group**	**Preintervention**	**Postintervention**	***F***	***P***
VIIT	0.83 ± 0.06	0.93 ± 0.02	60.635	< 0.001
MICT	0.87 ± 0.07	0.96 ± 0.03	48.897	< 0.001
Control group	0.83 ± 0.02	0.83 ± 0.02	0.010	0.921
*F*	3.066	145.958		
*P*	0.056	< 0.001		
General Inspection			
Time	*F =* 71.838, *P < * 0.001			
Group	*F =* 32.584, *P < * 0.001			
Time × Group	*F =*18.852, *P < * 0.001			

VIIT, variable intensity interval training; MICT, moderate-intensity continuous training.

shown in Table 5, no significant group differences in baseline RT were found (*F*_(2, 45)_ = 0.704, *P* = 0.500, ηp^2^ = 0.030), confirming equivalence across groups. After the intervention, RTs significantly decreased in both the VIIT and MICT groups (*P* < 0.001), with no significant changes in the control group (*P* = 0.733). Between-group comparisons of postintervention RTs revealed significant differences (*F*_(2, 45)_ = 8.005, *P* < 0.001, ηp^2^ = 0.262).

**Table 5:** Comparative analysis of stroop reaction times for the three groups.

**Group**	**Preintervention**	**Postintervention**	***F***	***P***
VIIT	795.29 ± 170.63	692.38 ± 123.71	29.218	< 0.001
MICT	746.17 ± 119.44	643.34 ± 99.24	29.175	< 0.001
Control group	792.60 ± 92.93	786.07 ± 79.89	0.118	0.733
*F*	0.704	8.005		
*P*	0.500	< 0.001		
General Inspection				
Time	*F* = 41.440, *P* < 0.001			
Group	*F* = 2.870, *P* = 0.067			
Time × Group	*F* = 8.535, *P* < 0.001			

VIIT, variable intensity interval training; MICT, moderate-intensity continuous training.

### 3.2 Behavioral results of inhibitory control during the 2-back task

As shown in Table 6, baseline ACC scores did not differ significantly across groups (*F*_(2, 45)_ = 1.181, *P* = 0.316, ηp^2^ = 0.050), suggesting equivalent initial performance. After intervention, the VIIT group showed a non-significant trend toward improved ACC (*P* = 0.072), whereas the MICT group demonstrated a significant increase in ACC (*P* < 0.001). The control group showed no significant change (*P* = 0.918). A significant between-group difference was observed postintervention (*F*_(2, 45)_ = 2.251, *P* = 0.009, ηp^2^ = 0.189).

**Table 6:** Comparative analysis of 2-BACK accuracy rates for three groups.

**Group**	**Preintervention**	**Postintervention**	***F***	***P***
VIIT	0.86 ± 0.08	0.90 ± 0.05	3.386	0.072
MICT	0.86 ± 0.07	0.92 ± 0.04	12.427	< 0.001
Control group	0.87 ± 0.05	0.87 ± 0.06	0.011	0.918
*F*	0.023	5.251		
*P*	0.978	0.009		
General inspection				
Time	*F* = 9.229, *P* = 0.004			
Group	*F* = 1.181, *P* = 0.316			
Time × Group	*F* = 3.297, *P* = 0.046			

VIIT, variable intensity interval training; MICT, moderate-intensity continuous training.

As shown in Table 7, no significant differences were observed in baseline RT among the three groups (*F*_(2, 45)_ = 0.434, *P* = 0.650, ηp^2^ = 0.019). However, both the VIIT and MICT groups exhibited significantly shorter RTs after the intervention (*P* < 0.001), whereas no significant change was observed in the control group (*P* = 0.347). Postintervention group comparisons indicated a significant group effect (*F*_(2, 45)_ = 7.343, *P* = 0.002, ηp^2^ = 0.246).

**Table 7:** Comparative analysis of 2-BACK reaction times for the three groups.

**Group**	**Preintervention**	**Postintervention**	***F***	***P***
VIIT	783.44 ± 151.97	664.38 ± 138.17	27.615	< 0.01
MICT	812.28 ± 104.85	653.62 ± 84.17	49.04	< 0.01
Control group	825.38 ± 129.64	803.83 ± 140.43	0.904	0.347
*F*	0.434	7.343		
*P*	0.650	0.002		
General inspection				
Time	*F* = 58.158, *P* < 0.001			
Group	*F* = 2.837, *P* = 0.069			
Time × Group	*F* = 9.701, *P* < 0.001			

VIIT, variable intensity interval training; MICT, moderate-intensity continuous training.

### 3.3 Behavioral results of inhibitory control during the more-odd shifting task

As shown in Table 8, no significant differences in baseline ACC were found among the three groups (*F*_(2, 45)_ = 0.959, *P* = 0.391, ηp^2^ = 0.041), indicating comparable baseline levels. Postintervention, both the VIIT and MICT groups presented significantly improved ACC values compared with the pretest values (*P* < 0.01), whereas the control group showed no significant change (*P* = 0.585). Between-group comparisons after the intervention revealed a significant effect (*F*_(2, 45)_ = 23.111, *P* < 0.01, ηp^2^ = 0.507).

**Table 8:** Comparative analysis of more accuracy rates for three groups.

**Group**	**Preintervention**	**Postintervention**	***F***	***P***
VIIT	0.82 ± 0.08	0.93 ± 0.03	84.458	< 0.01
MICT	0.84 ± 0.04	0.91 ± 0.03	35.667	< 0.01
Control group	0.85 ± 0.04	0.85 ± 0.04	0.303	0.585
*F*	0.959	23.111		
*P*	0.391	< 0.01		
General inspection				
Time	*F* = 82.295, *P* < 0.01			
Group	*F* = 1.731, *P* = 0.189			
Time × Group	*F* = 19.066, *P* < 0.01			

VIIT, variable intensity interval training; MICT, moderate-intensity continuous training.

As shown in Table 9, the baseline RT did not differ significantly among groups (*F*_(2, 45)_ = 2.219, *P* = 0.121, ηp^2^ = 0.090). Postintervention, RT in the VIIT group significantly decreased (*P* < 0.001), as did RT in the MICT group (*P* < 0.001), whereas no significant change in RT was observed in the control group (*P* = 0.207). However, the between-group difference in RT after intervention did not reach statistical significance (*F*_(2, 45)_ = 2.060, *P* = 0.139, ηp^2^ = 0.084).

**Table 9:** Comparative analysis of more reaction times for three groups.

**Group**	**Preintervention**	**Postintervention**	***F***	***P***
VIIT	1143.83 ± 161.41	927.9 ± 176.48	58.105	< 0.001
MICT	1050.12 ± 149.51	890.6 ± 191.28	31.713	< 0.001
Control group	1045.06 ± 136.14	1008.76 ± 131.40	1.642	0.207
*F*	2.219	2.060		
*P*	0.121	0.139		
General Inspection				
Time	*F* = 70.426, *P* < 0.01			
Group	*F* = 0.913, *P* = 0.409			
Time × Group	*F* = 10.516, *P* < 0.01			

VIIT, variable intensity interval training; MICT, moderate-intensity continuous training.

### 3.4 Prefrontal hemodynamic responses during the stroop task

**Figure 4:**
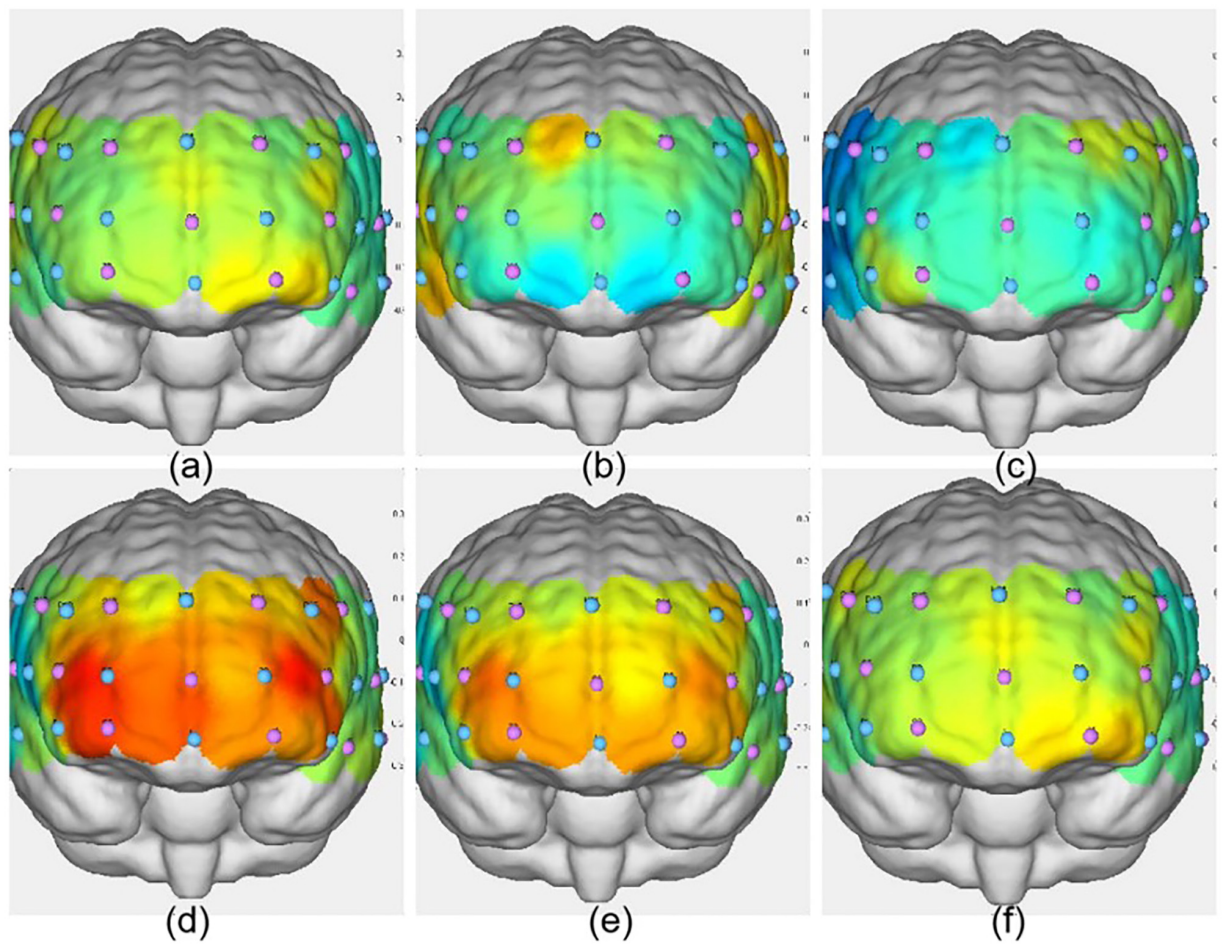
Activation maps of the frontal lobe during the three groups' Stroop tasks. **(a)** VIIT group pre-test; **(b)** MICT group pre-test; **(c)** control group pre-test; **(d)** VIIT group post-test; **(e)** MICT group post-test; **(f)** Control group post-test. The greater the ΔHbO_2_ value, the warmer the color; the smaller the ΔHbO_2_ value, the cooler the color.

**Figure 5:**
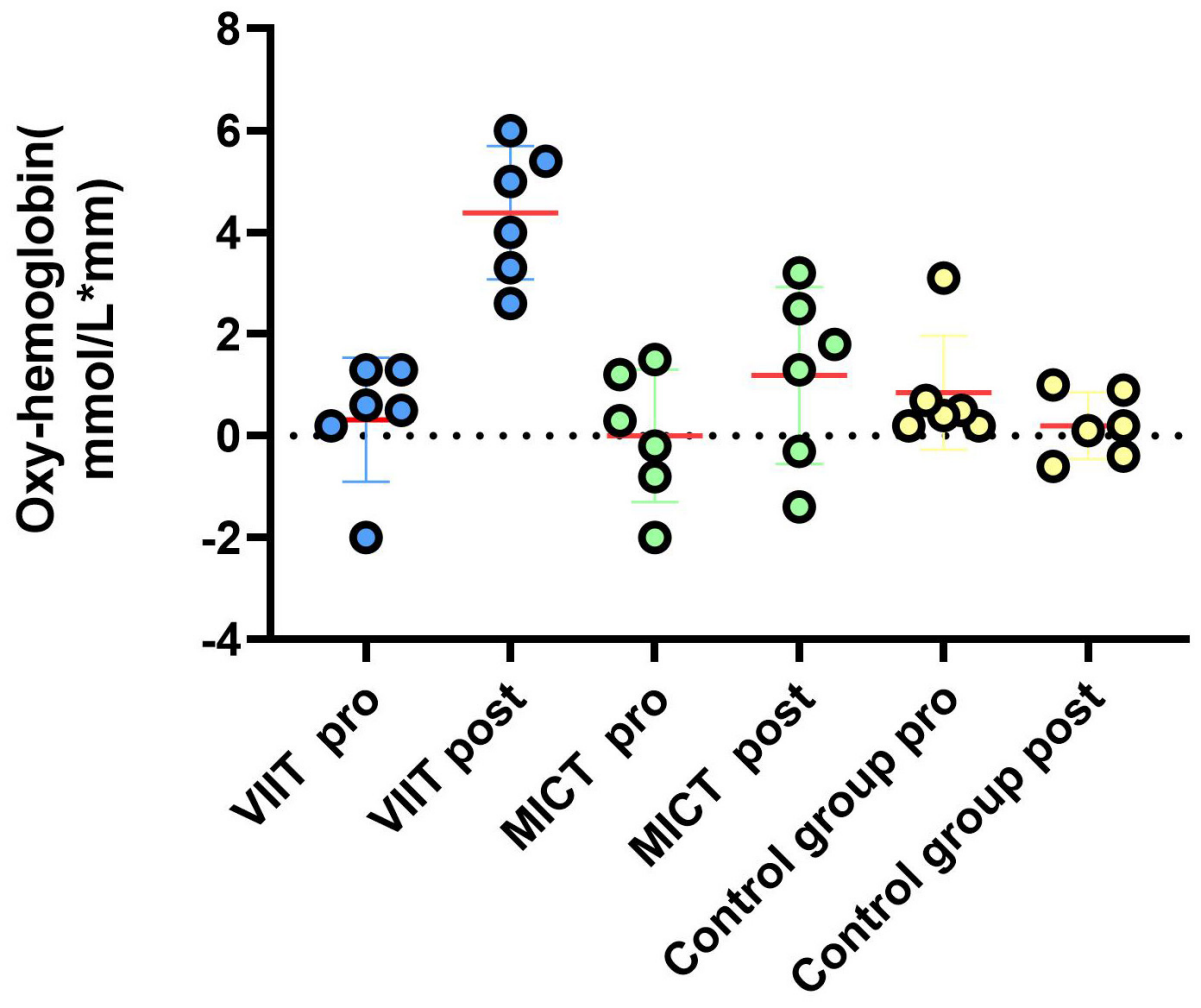
Prefrontal hemodynamic responses during the Stroop task. A comparison of the ΔHbO_2_ results between the VIIT, MICT, and Control groups before and after performing the Stroop task (channels 41, 42, 44, 26, 27, 4): reveals significant changes in the VIIT group.

As shown in Table 10, postintervention analysis revealed significant increases in ΔHbO_2_ in the VIIT group across multiple channels: Channel 41: *F*_(1, 45)_ = 6.651, *P* = 0.013, ηp^2^ = 0.129; Channel 42: *F*_(1, 45)_ = 14.017, *P* < 0.001, ηp^2^ = 0.238; Channel 44: *F*_(1, 45)_ = 7.106, *P* = 0.011, ηp^2^ = 0.136; Channel 26: *F*_(1, 45)_ = 14.533, *P* < 0.001, ηp^2^ = 0.244; Channel 27: *F*_(1, 45)_ = 14.064, *P* < 0.001, ηp^2^ = 0.238; Channel 4: *F*_(1, 45)_ = 11.505, *P* = 0.001, ηp^2^ = 0.204. In the MICT group, significant increases in ΔHbO_2_ were observed in Channel 42: *F*_(1, 45)_ = 8.400, *P* = 0.006, ηp^2^ = 0.157; Channel 27: *F*_(1, 45)_ = 5.855, *P* = 0.020, ηp^2^ = 0.115; and Channel 4: *F*_(1, 45)_ = 4.751, *P* = 0.035, ηp^2^ = 0.095. Figures 4, 5 illustrate these results. Notably, postintervention comparisons between the VIIT group and the control group revealed significant ΔHbO_2_ increases in Channel 42 (*P* = 0.007), Channel 44 (*P* < 0.001), and Channel 26 (*P* = 0.006).

**Table 10:** ΔHbO_2_ data (Mean ± SD) of inhibitory function in the experimental group and control group.

**Brain area**	**Passageway**	**VIIT (*****n*** = **17)**		**MICT (*****n*** = **17)**		**Control group (*****n*** = **17)**	
		**Pre**	**Post**	**Pre**	**Post**	**Pre**	**Post**
D	22	0.6 ± 6.5	4.3 ± 17.3	0.6 ± 5.9	−0.9 ± 4.8	0.8 ± 6.4	1.0 ± 11.5
L	24	−0.3 ± 5.8	3.1 ± 13.4	0.8 ± 5.1	−2.7 ± 6.1	0.3 ± 6.1	−0.5 ± 5.6
P	32	−0.1 ± 5.8	−0.2 ± 10.9	−0.5 ± 5.1	1.1 ± 4.2	0.4 ± 6.6	0.4 ± 5.3
E	38	−0.7 ± 5.6	3.9 ± 13.5	2.3 ± 8.7	3.2 ± 12.9	−0.1 ± 6.5	0.9 ± 5.1
C	39	−0.1 ± 3.9	3.3 ± 17.6	3.0 ± 9.2	0.9 ± 7.2	3.7 ± 8.9	−0.4 ± 3.9
	41	1.3 ± 4.5	6.0 ± 3.4^*^	1.2 ± 6.9	−1.4 ± 8.1	3.1 ± 7.8	0.9 ± 5.2
	42	0.6 ± 1.8	5.0 ± 2.8^*^#	−0.8 ± 2.2	2.5 ± 2.4^*^	0.2 ± 2.3	0.2 ± 2.3
	44	0.5 ± 2.4	2.6 ± 1.7^*^#	0.3 ± 2.5	−0.3 ± 1.9^*^	0.2 ± 2.5	−0.4 ± 2.9
	45	2.2 ± 6.7	−0.9 ± 5.0	2.2 ± 3.4	3.5 ± 8.4	1.5 ± 4.9	−0.4 ± 5.3
	47	3.7 ± 11.4	0.9 ± 10.9	1.0 ± 7.6	4.5 ± 14.0	1.2 ± 8.2	0.7 ± 4.5
	6	−2.6 ± 8.3	−1.8 ± 10.6	−3.9 ± 5.5	−1.4 ± 11.2	2.9 ± 10.8	0.5 ± 8.6
	8	0.2 ± 7.4	0.8 ± 13.0	−0.3 ± 5.1	−1.7 ± 6.8	2.9 ± 7.9	−0.9 ± 10.0
	11	0.0 ± 8.3	−0.4 ± 8.5	−0.5 ± 4.4	0.0 ± 6.0	2.8 ± 7.3	−1.4 ± 8.7
	21	2.5 ± 6.8	2.7 ± 13.2	0.2 ± 5.6	0.3 ± 9.5	2.2 ± 7.1	1.5 ± 9.5
	23	1.9 ± 9.0	2.6 ± 11.4	1.4 ± 7.1	−0.6 ± 5.6	5.1 ± 10.6	−0.5 ± 8.2
	25	−1.8 ± 6.0	−1.4 ± 9.5	0.5 ± 5.2	1.6 ± 8.0	0.9 ± 8.3	−0.2 ± 10.2
	26	1.3 ± 1.6	4.0 ± 1.6^*^#	1.5 ± 2.2	1.8 ± 3.1	0.5 ± 3.1	1.0 ± 2.8
	27	−2.0 ± 6.9	3.3 ± 6.2	−0.2 ± 3.0	3.2 ± 2.3	0.4 ± 7.7	0.1 ± 2.4
	28	−0.8 ± 5.9	−0.2 ± 7.7	−0.3 ± 5.2	−0.1 ± 5.2	1.1 ± 5.6	−1.0 ± 5.9
	40	0.1 ± 5.5	−0.9 ± 10.4	1.4 ± 6.1	−0.7 ± 4.6	3.5 ± 8.0	0.5 ± 5.7
	43	0.6 ± 6.4	2.5 ± 8.2	0.6 ± 3.8	1.0 ± 4.1	0.3 ± 8.1	−0.1 ± 4.1
V	4	0.2 ± 4.3	5.4 ± 3.5^*^	−2.0 ± 7.1	1.3 ± 7.4^*^	0.7 ± 7.8	−0.6 ± 10.4
L	5	1.8 ± 5.2	0.8 ± 8.3	0.8 ± 7.5	−5.1 ± 10.1	−1.0 ± 7.1	−2.3 ± 12.4
P	19	4.2 ± 6.1	0.2 ± 8.0	2.0 ± 11.9	−3.9 ± 7.7	3.3 ± 11.0	0.4 ± 9.9
F	29	1.4 ± 7.1	−0.1 ± 14.1	1.7 ± 5.0	−1.1 ± 7.2	1.7 ± 7.1	−0.6 ± 10.5
C	31	2.9 ± 6.5	1.6 ± 9.6	−0.1 ± 6.0	−0.6 ± 7.8	−1.2 ± 8.2	−0.5 ± 6.5
	37	1.8 ± 4.6	2.6 ± 11.5	3.2 ± 7.5	−1.1 ± 6.1	3.5 ± 6.9	0.1 ± 7.4
	46	1.0 ± 6.8	1.2 ± 8.0	1.2 ± 5.8	−0.5 ± 4.1	0.6 ± 5.2	1.4 ± 6.5
O	7	−3.9 ± 9.5	−1.0 ± 11.8	−3.5 ± 7.5	−4.0 ± 10.9	0.3 ± 9.5	−1.3 ± 8.2
F	9	−2.9 ± 8.3	−2.1 ± 7.5	−2.7 ± 7.6	−3.6 ± 8.3	−0.2 ± 10.4	−2.8 ± 9.6
C	10	−0.5 ± 7.7	−2.1 ± 10.3	−2.1 ± 4.4	−0.5 ± 9.3	2.4 ± 7.0	−3.7 ± 10.5

^*^ indicates that there is a difference before and after in the group, *P* < 0.05; # indicates that there is a difference between the group and the back test between the two groups of the control group, *P* < 0.05. DLPFC, dorsolateral prefrontal cortex; VLPFC, ventrolateral prefrontal cortex; OFC, orbitofrontal cortex.

### 3.5 Prefrontal hemodynamic responses during the 2-back task

**Figure 6:**
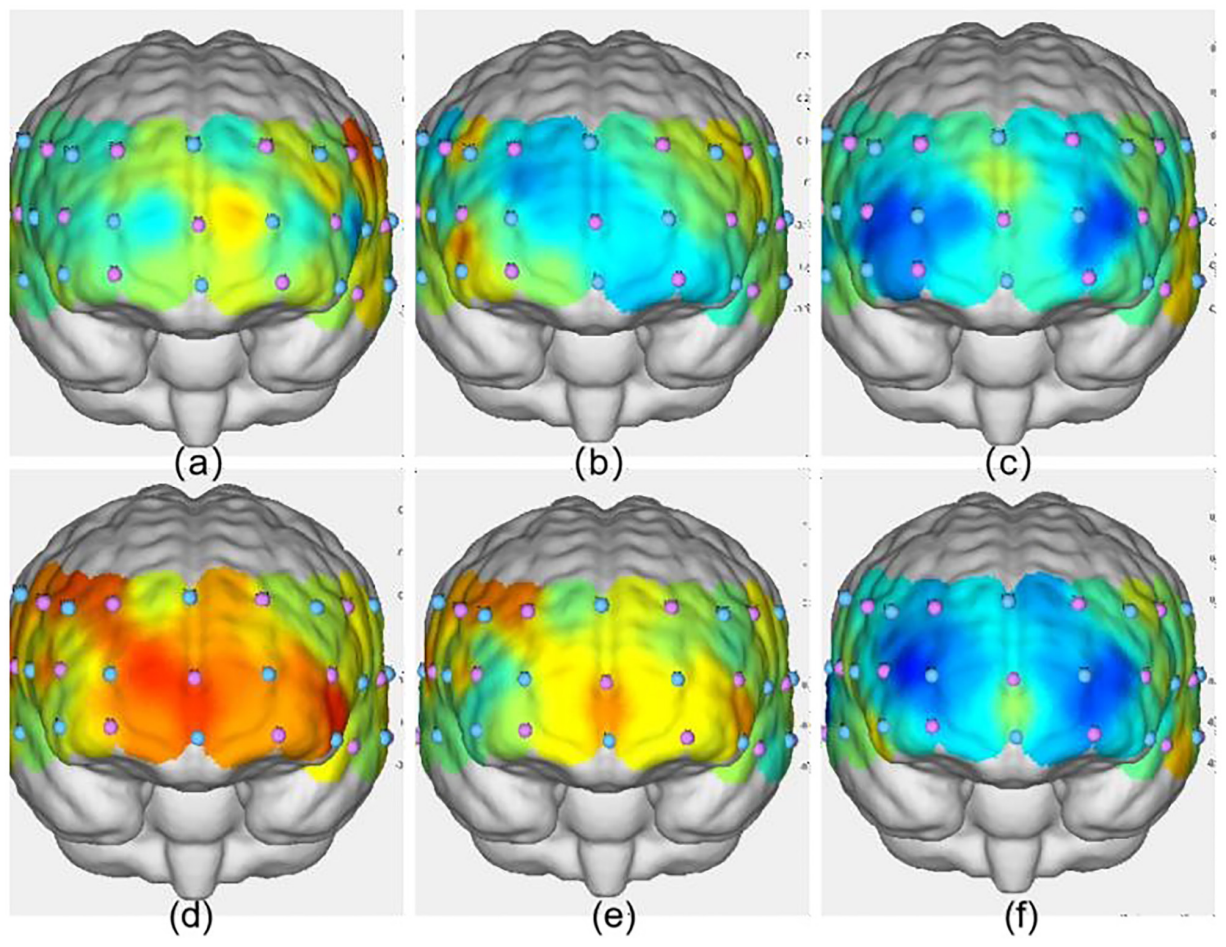
Activation maps of the frontal lobe during the three groups' 2-back tasks. **(a)** VIIT group pre-test; **(b)** MICT group pre-test; **(c)** control group pre-test; **(d)** VIIT group post-test; **(e)** MICT group post-test; **(f)** Control group post-test. The greater the ΔHbO_2_ value, the warmer the color; the smaller the ΔHbO_2_ value, the cooler the color.

**Figure 7:**
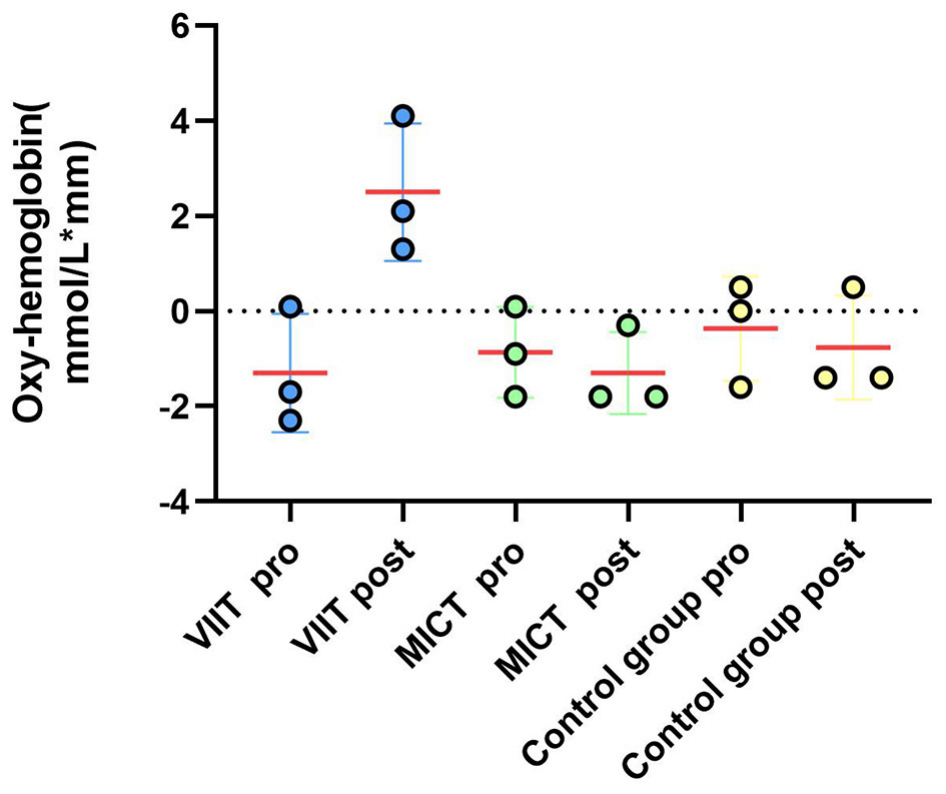
Prefrontal hemodynamic responses during the 2-back task: a comparison of the ΔHbO_2_ results between the VIIT, MICT, and Control groups before and after performing the 2-back task (channels 40, 43, 41) shows significant changes in the VIIT group.

As shown in Table 11, significant increases in ΔHbO_2_ were observed in the VIIT group postintervention in the following channels: Channel 40: *F*_(1, 45)_ = 8.846, *P* = 0.005, ηp^2^ = 0.164; Channel 43: *F*_(1, 45)_ = 10.116, *P* = 0.003, ηp^2^ = 0.184; and Channel 41: *F*_(1, 45)_ = 12.978, *P* < 0.001, ηp^2^ = 0.224. In the MICT group, a significant increase was observed in Channel 40: *F*_(1, 45)_ = 4.396, *P* = 0.042, ηp^2^ = 0.089; these results are visualized in Figures 6, 7.

**Table 11:** ΔHbO_2_ data (Mean ± SD) of refresh function in the experimental group and control group.

**Brain area**	**Passageway**	**VIIT (*****n*** = **17)**		**MICT (*****n*** = **17)**		**Control group (*****n*** = **17)**	
		**Pre**	**Post**	**Pre**	**Post**	**Pre**	**Post**
D	22	4.0 ± 10.3	−1.4 ± 8.3	0.2 ± 6.9	0.9 ± 5.6	2.9 ± 7.3	−2.4 ± 8.2
L	24	0.4 ± 8.6	−0.8 ± 8.1	0.1 ± 4.6	−0.0 ± 3.8	3.0 ± 7.4	−5.0 ± 10.1
P	32	−1.1 ± 8.9	−0.9 ± 9.5	−1.6 ± 4.0	−1.0 ± 4.6	−2.6 ± 8.6	0.4 ± 6.3
E	38	5.0 ± 10.4	1.2 ± 7.7	−1.3 ± 13.2	−3.4 ± 7.4	3.4 ± 8.5	1.5 ± 5.9
C	39	1.4 ± 6.4	−1.5 ± 7.1	0.8 ± 5.1	−1.5 ± 6.4	4.1 ± 7.5	1.5 ± 4.7
	41	0.1 ± 3.2	4.1 ± 5.7^*^	0.1 ± 3.6	−0.3 ± 3.2	0.5 ± 6.3	0.5 ± 6.4
	42	17.0 ± 6.3	17.0 ± 6.4	16.0 ± 3.4	16.0 ± 2.9	18.0 ± 5.2	18.0 ± 4.5
	44	0.1 ± 5.7	−3.4 ± 8.5	0.0 ± 3.6	−3.0 ± 4.9	−1.9 ± 5.9	−0.1 ± 9.8
	45	0.0 ± 8.8	−1.3 ± 6.8	0.4 ± 4.9	−2.5 ± 5.7	1.3 ± 7.6	0.3 ± 7.2
	47	−1.7 ± 15.3	−0.6 ± 8.0	0.6 ± 6.2	0.9 ± 7.5	1.8 ± 11.6	2.6 ± 9.6
	6	−4.5 ± 7.1	−4.7 ± 9.0	−4.8 ± 10.6	−4.8 ± 7.1	−5.6 ± 11.5	−3.8 ± 7.8
	8	−1.5 ± 11.1	−3.9 ± 10.4	−0.5 ± 3.7	−3.7 ± 5.2	−0.7 ± 7.2	1.1 ± 9.4
	11	−5.2 ± 7.9	−6.1 ± 10.8	−3.0 ± 5.5	−3.4 ± 4.7	−0.4 ± 9.3	−1.0 ± 8.3
	21	−1.8 ± 8.9	−3.1 ± 10.1	−0.6 ± 8.0	−2.0 ± 7.0	−1.7 ± 7.3	−6.1 ± 29.0
	23	−0.6 ± 7.9	−4.5 ± 7.8	1.2 ± 4.9	−1.7 ± 5.5	−0.9 ± 8.9	−2.3 ± 9.2
	25	−4.9 ± 10.4	−5.3 ± 7.0	−1.0 ± 4.0	−3.8 ± 7.2	−4.6 ± 10.6	−5.9 ± 12.2
	26	−4.1 ± 6.3	−7.1 ± 7.2	0.1 ± 4.6	−1.8 ± 3.7	−4.9 ± 6.9	−3.2 ± 7.2
	27	−3.1 ± 7.2	−6.4 ± 8.9	0.7 ± 5.0	−2.9 ± 4.3	−4.7 ± 4.9	−2.6 ± 6.2
	28	−0.2 ± 4.8	−3.3 ± 8.2	0.3 ± 3.7	−2.8 ± 4.8	−0.8 ± 5.0	−2.2 ± 5.8
	40	−1.7 ± 4.1	2.1 ± 3.3^*^	−0.9 ± 3.5	1.8 ± 4.5^*^	−0.0 ± 5.4	−1.4 ± 4.4
	43	−2.3 ± 3.6	1.3 ± 6.0^*^	−1.8 ± 3.6	−1.8 ± 3.3	1.6 ± 3.8	−1.4 ± 3.5
V	4	−1.0 ± 11.3	−4.7 ± 12.0	−3.8 ± 12.2	−4.8 ± 6.4	−2.3 ± 14.5	−6.0 ± 10.5
L	5	1.0 ± 11.4	−3.7 ± 10.3	−4.2 ± 7.2	−2.9 ± 6.8	−3.7 ± 7.0	−5.7 ± 11.7
P	19	4.9 ± 11.1	0.4 ± 8.8	−3.1 ± 6.6	1.3 ± 4.9	0.6 ± 12.3	−3.2 ± 9.9
F	29	−4.9 ± 11.2	−4.9 ± 10.6	−2.2 ± 6.7	−3.6 ± 6.4	−2.5 ± 8.9	−0.6 ± 6.3
C	31	−1.2 ± 14.5	−2.6 ± 7.7	−3.1 ± 6.8	−0.8 ± 3.9	−1.6 ± 13.8	2.0 ± 8.5
	37	3.4 ± 9.8	2.3 ± 11.0	1.7 ± 8.0	−1.6 ± 5.9	4.7 ± 7.5	−0.9 ± 11.2
	46	1.2 ± 8.5	−1.4 ± 7.2	−1.1 ± 6.9	−2.4 ± 5.7	0.3 ± 6.2	1.5 ± 7.7
O	7	−6.0 ± 9.9	−5.6 ± 9.3	−4.0 ± 8.4	−4.7 ± 9.7	−4.8 ± 11.5	−3.9 ± 11.1
F	9	−7.7 ± 8.0	−7.0 ± 9.8	−2.7 ± 6.0	−3.8 ± 7.6	−5.3 ± 11.2	−3.3 ± 12.6
C	10	−6.4 ± 10.4	−7.0 ± 7.1	−2.6 ± 7.4	−2.3 ± 6.2	−2.9 ± 9.7	−3.6 ± 5.9

^*^ indicates that there is a difference before and after in the group, *P* < 0.05; DLPFC, dorsolateral prefrontal cortex; VLPFC, ventrolateral prefrontal cortex; OFC, orbitofrontal cortex.

### 3.6 Prefrontal hemodynamic responses during the more-odd shifting task

**Figure 8:**
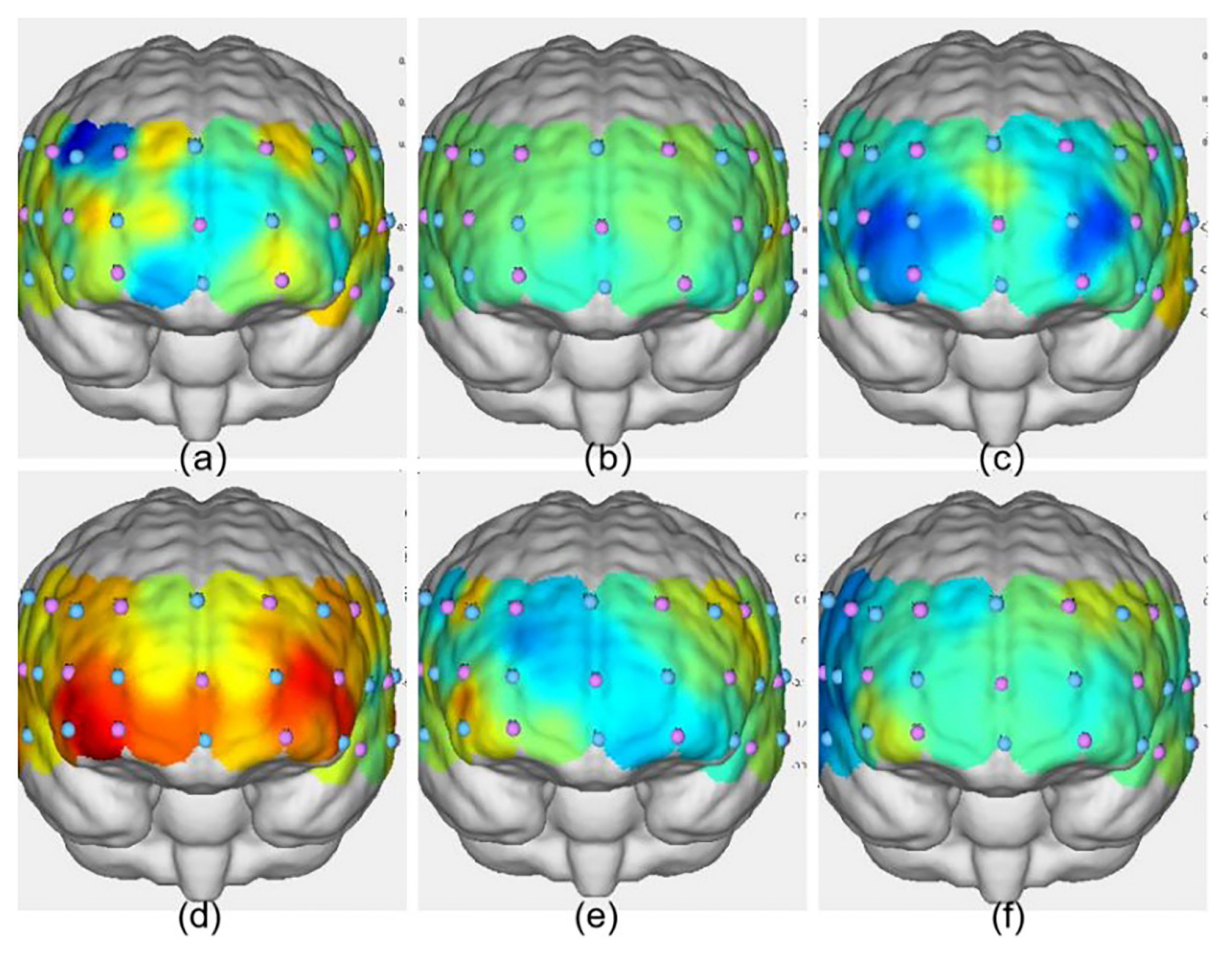
Activation maps of the frontal lobe during the three groups' more-odd shifting tasks. **(a)** VIIT group pre-test; **(b)** MICT group pre-test; **(c)** control group pre-test; **(d)** VIIT group post-test; **(e)** MICT group post-test; **(f)** Control group post-test. The greater the ΔHbO_2_ value, the warmer the color; the smaller the ΔHbO_2_ value, the cooler the color.

**Figure 9:**
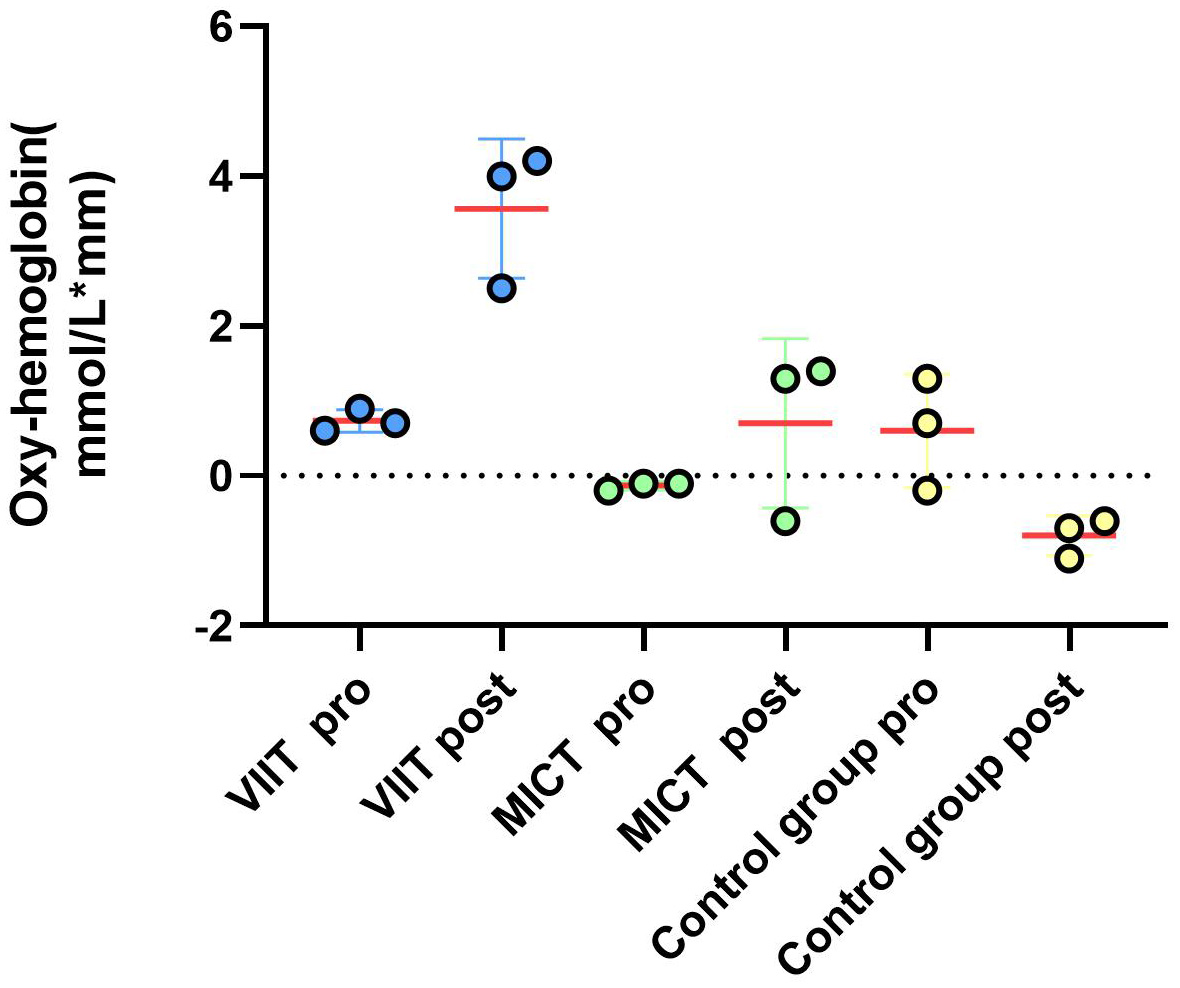
Prefrontal hemodynamic responses during the more-odd shifting task: a comparison of the ΔHbO_2_ results between the VIIT, MICT, and Control groups before and after performing the more-odd shifting task (channels 42, 44, 4) indicates significant changes in the VIIT group.

As shown in Table 12, following the intervention, the VIIT group presented significant increases in ΔHbO_2_ in Channel 42: *F*_(1, 45)_ = 6.593, *P* = 0.014, ηp^2^ = 0.128; Channel 44: *F*_(1, 45)_ = 6.521, *P* = 0.014, ηp^2^ = 0.127; and Channel 4: *F*_(1, 45)_ = 4.504, *P* = 0.039, ηp^2^ = 0.091. The results are presented in Figures 8, 9.

**Table 12:** ΔHbO_2_ data (Mean ± SD) of shift function in the experimental group and control group.

**Brain area**	**Passageway**	**VIIT (*****n*** = **17)**		**MICT (n** = **17)**		**Control group (*****n*** = **17)**	
							
D	22	0.1 ± 11.2	−0.1 ± 8.2	−1.7 ± 5.1	−1.8 ± 6.3	1.4 ± 6.7	0.0 ± 7.8
L	24	−3.9 ± 10.1	4.3 ± 10.3	−2.5 ± 6.6	−2.8 ± 6.5	−4.0 ± 9.1	−0.3 ± 6.0
P	32	−1.3 ± 8.1	−1.8 ± 8.0	−0.6 ± 4.5	−1.7 ± 4.9	−0.8 ± 6.6	−1.7 ± 5.3
E	38	−3.5 ± 9.2	−2.5 ± 8.8	2.4 ± 11.9	−1.9 ± 7.9	3.1 ± 12.5	4.8 ± 10.7
C	39	−0.6 ± 5.3	−0.8 ± 7.6	−2.1 ± 6.2	−1.1 ± 7.2	0.7 ± 7.0	1.1 ± 5.1
	41	−0.7 ± 6.9	1.4 ± 8.5	−2.0 ± 4.2	0.1 ± 9.7	−0.8 ± 6.4	2.2 ± 10.0
	42	0.6 ± 1.8	4.2 ± 4.2^*^#	−0.2 ± 1.9	1.4 ± 3.8	0.7 ± 1.8	−0.7 ± 6.9
	44	0.7 ± 2.0	2.5 ± 2.9^*^#	−0.1 ± 2.0	−0.6 ± 1.7^*^	−0.2 ± 2.1	−0.6 ± 1.7
	45	0.9 ± 5.6	−1.4 ± 7.6	−0.1 ± 3.2	−1.1 ± 6.8	0.1 ± 7.4	−1.1 ± 5.3
	47	4.4 ± 11.3	1.6 ± 11.8	−0.2 ± 3.7	−3.8 ± 5.7	2.3 ± 11.0	−1.2 ± 5.7
	6	−4.2 ± 8.2	−1.9 ± 10.0	−2.4 ± 7.4	−4.9 ± 8.0	−0.4 ± 10.2	−5.1 ± 8.9
	8	−3.9 ± 9.1	−1.6 ± 8.0	−1.9 ± 8.2	−5.3 ± 9.9	−3.1 ± 11.5	−3.8 ± 9.0
	11	−2.5 ± 9.2	−2.0 ± 8.0	−2.3 ± 7.8	−0.2 ± 7.3	−0.4 ± 9.0	−7.6 ± 10.6
	21	−3.0 ± 9.5	0.0 ± 11.6	−3.9 ± 4.5	−2.5 ± 5.0	−1.9 ± 6.4	−5.3 ± 11.0
	23	−1.4 ± 8.8	0.6 ± 8.2	−3.4 ± 7.2	−3.0 ± 6.9	−2.8 ± 8.6	−1.8 ± 8.0
	25	−1.5 ± 9.0	−2.5 ± 6.4	0.0 ± 5.8	−1.7 ± 7.6	−2.3 ± 8.4	−2.1 ± 10.7
	26	−3.4 ± 5.3	0.8 ± 6.5	0.3 ± 6.7	−3.8 ± 4.6	−2.3 ± 8.3	−3.2 ± 6.6
	27	−2.4 ± 9.0	−1.4 ± 9.8	−0.5 ± 6.9	−1.5 ± 5.2	−3.8 ± 8.6	−4.1 ± 5.6
	28	−1.3 ± 5.7	−1.5 ± 7.4	1.4 ± 6.4	−1.1 ± 7.9	−1.2 ± 6.0	−1.4 ± 6.1
	40	−2.7 ± 5.2	0.6 ± 8.6	−0.2 ± 7.0	−1.4 ± 5.8	−2.0 ± 7.5	−2.4 ± 4.7
	43	−2.3 ± 5.7	−2.8 ± 5.2	−1.1 ± 6.5	−1.6 ± 5.9	−3.6 ± 6.4	−0.6 ± 5.9
V	4	0.9 ± 3.8	4.0 ± 5.3^*^	−0.1 ± 4.4	1.3 ± 7.4	1.3 ± 3.8	−1.1 ± 5.4
L	5	−1.7 ± 8.5	−4.9 ± 9.7	−0.8 ± 7.3	−5.2 ± 10.1	−1.8 ± 15.0	−1.5 ± 10.0
P	19	0.6 ± 9.9	0.1 ± 10.1	−2.2 ± 7.8	−3.6 ± 14.7	−0.4 ± 8.4	−1.9 ± 7.3
F	29	−3.8 ± 8.4	−1.2 ± 10.7	−2.7 ± 6.1	−1.3 ± 4.4	−3.5 ± 8.3	−3.7 ± 9.8
C	31	−1.9 ± 6.6	−1.9 ± 5.6	−2.7 ± 5.9	−3.2 ± 5.6	−3.6 ± 8.7	−1.5 ± 7.6
	37	0.5 ± 6.2	−0.3 ± 7.3	2.0 ± 8.4	0.2 ± 5.9	0.2 ± 8.7	1.8 ± 7.9
	46	−0.6 ± 5.1	−0.2 ± 10.0	−0.7 ± 5.3	−2.0 ± 6.9	−3.3 ± 6.5	−1.7 ± 8.8
O	7	−4.3 ± 10.8	0.8 ± 17.1	−4.3 ± 7.2	−4.3 ± 8.9	−3.1 ± 11.3	−9.7 ± 14.7
F	9	−6.0 ± 7.3	−4.6 ± 7.3	−3.8 ± 6.8	−3.1 ± 9.9	−3.9 ± 7.4	−5.4 ± 9.8
C	10	−3.7 ± 7.4	−2.7 ± 10.9	−4.0 ± 7.4	−2.7 ± 8.2	−2.4 ± 10.6	−4.2 ± 8.4

^*^ indicates that there is a difference before and after in the group, *P* < 0.05; # indicates that there is a difference between the group and the back test between the two groups of the control group, *P* < 0.05. DLPFC, dorsolateral prefrontal cortex; VLPFC, ventrolateral prefrontal cortex; OFC, orbitofrontal cortex.

## 4 Discussion

This study demonstrated that exercise interventions significantly improved inhibitory control, which is consistent with prior research findings on the cognitive benefits of physical activity (Zhang et al., 2023). From a neurophysiological perspective, the enhancement of inhibitory function through exercise can be explained by three major mechanisms:

First, neuroendocrine regulation: physical activity significantly elevates the plasma levels of norepinephrine (NE) and cortisol (Tsai et al., 2014), while also promoting the release of central nervous system neurotransmitters such as dopamine (DA) and endorphins (McMorris and Hale, 2012). The combined action of these neuromodulators enhances catecholamine activity, which in turn strengthens attentional vigilance by modulating circuits between the prefrontal cortex and limbic system (McMorris et al., 2008).

Second, cerebral blood flow optimization: exercise improves cerebrovascular regulation, increases cerebral blood flow (CBF), and enhances oxygenation in the brain (Liu et al., 2023), These effects ensure better delivery of oxygenated hemoglobin to active neurons, thereby optimizing the neural metabolic efficiency required during cognitive task performance.

Third, neural network remodeling: complex physical tasks continuously demand attentional resources and cognitive control, which reinforces the functional connectivity of key nodes in executive control networks, such as the anterior cingulate cortex (ACC) and DLPFC (Wang et al., 2024), These neuroplastic changes may underlie the superior performance observed in the exercise groups during inhibition and attention-related tasks.

As a classical paradigm for assessing inhibitory control within executive function, the Stroop task is widely used to measure an individual's ability to resolve conflicting information under time pressure. Neuroimaging studies have confirmed that successful Stroop performance relies primarily on the coordinated activity of the fronto-striatal circuitry, with marked activation in the VLPFC, DLPFC, and FPA (Yanagisawa et al., 2010). Among these, the DLPFC acts as a core node of the executive network and plays a crucial role in top-down attentional control during selective attention in Stroop processing (Causse et al., 2018; Vanderhasselt et al., 2006).

In this study, significant increases in ΔHbO_2_ were observed in key regions—including the DLPFC (channels 41, 42, 44), FPA (channels 26, 27), and VLPFC (channel 4)—following the VIIT intervention. Compared with the MICT group, the VIIT group presented greater prefrontal cortical activation during the Stroop task. This finding may be attributed to the following unique physiological characteristics of VIIT: (1) its high-intensity nature elicits greater metabolic demands, rapidly increasing the heart rate and metabolic rate, thereby triggering stronger cerebrovascular responses (Yu et al., 2025); and (2) the EPOC effect induced by VIIT may prolong metabolic activation in the brain, resulting in sustained neural activation during cognitive tasks (Vanderhasselt et al., 2006; Liu et al., 2025). In contrast, although MICT also facilitated cognitive improvements, its relatively lower intensity and stable metabolic stimulation may have induced a milder degree of neural activation.

Both the VIIT and MICT interventions significantly shortened reaction times in the 2-back task compared with those in the control group. From a physiological standpoint, both the high-intensity intermittent stimulation of VIIT and the continuous moderate-intensity training of MICT likely enhanced cognitive function by increasing cerebral blood flow and improving neuroenergetic efficiency (Yu et al., 2025). The non-significant trend of ACC improvement in the VIIT group, in contrast to the significant improvement observed in the MICT group, may be explained by the dose-dependent effects of neuromodulators on prefrontal function, particularly dopamine. Low doses of dopamine D1 agonists improve working memory-related behavior, but high doses eliminate the improvement, thus yielding an ‘inverted-U' dose-response curve. While VIIT may be more metabolically efficient on a per-unit-time basis, the consistent stimulation pattern of MICT can also generate robust cognitive benefits (Marcourt et al., 2025). Despite their differences in exercise intensity, metabolic profiles, and physiological responses (Çakir et al., 2025), VIIT and MICT may be equally effective in enhancing cognitive function.

The updating function primarily involves two key processes: the maintenance of short-term memory and the execution of cognitive control. In the 2-back task, VIIT elicited a significantly greater increase in ΔHbO_2_ compared with MICT (Zhang et al., 2023). Specifically, VIIT induced marked increases in ΔHbO_2_ within the dorsolateral prefrontal cortex (channels 40, 43, 41), whereas MICT-related activation was predominantly observed in the frontopolar area (channel 40). The frontopolar cortex is involved in several higher-order cognitive processes, including information encoding and retrieval, problem solving, memory access during task execution, and complex reasoning (Dong et al., 2019), Notably, this region is strongly activated during working memory and complex cognitive-motor tasks—functions that are highly aligned with the underlying mechanisms of updating processes (Emch et al., 2019; Wible et al., 2009; Milot et al., 2018; Woolgar et al., 2015). Based on these findings, the present study proposes that activation of the frontopolar cortex may serve as a critical neural substrate through which exercise enhances updating ability.

Both MICT and VIIT interventions also significantly reduced reaction time in the task-switching condition. From a neurophysiological perspective, exercise interventions can enhance key hemodynamic parameters of the middle cerebral artery (MCA), particularly peak systolic velocity (PSV), which has been shown to be positively associated with improvements in cognitive function (Oliva et al., 2023), From an exercise physiology standpoint, both VIIT and MICT are effective in improving cardiorespiratory fitness (CRF) and promoting the release of brain-derived neurotrophic factor (BDNF), thereby enhancing neuroplasticity (Marcourt et al., 2025). Specifically, rapid alternating high-intensity stimulation of VIIT may be particularly effective in elevating the heart rate and metabolic rate, leading to more robust cerebrovascular responses.

This study further revealed that the VIIT intervention led to significantly increased ΔHbO_2_ in prefrontal brain regions, including the dorsolateral prefrontal cortex (channels 42, 44) and ventrolateral prefrontal cortex (channel 4). Physiologically, VIIT enhances cerebral vasodilation by rapidly increasing heart rate variability (HRV) and metabolic demand, thus improving oxygen delivery in target brain regions (Oliva et al., 2023). Additionally, the EPOC effect induced by VIIT may sustain elevated brain metabolism over time, potentially optimizing cognitive performance through enhanced neurotransmitter release and synaptic plasticity. In contrast, although MICT can improve basic cognitive functions, its relatively lower intensity may not generate sufficient metabolic and neuroendocrine stimulation to surpass the activation threshold necessary for robust prefrontal cortex engagement. Some studies have suggested that high-intensity exercise may induce stronger hemodynamic responses and neuroendocrine changes, leading to more profound modulation of prefrontal cortex function. This provides theoretical support for explaining why VIIT demonstrated stronger activation patterns across multiple tasks and brain regions compared to MICT.

## 5 Conclusion

VIIT and MICT significantly enhance the executive function of male college students, as indicated by improvements in ACC and RT. VIIT markedly increases the concentration of ΔHbO_2_ in the prefrontal cortex during tasks. While MICT also increases ΔHbO_2_ in certain channels, its overall effect is less pronounced and does not match the activation intensity of VIIT. The channels activated by VIIT are mainly in the frontopolar (channels 26, 27, 40, 43), dorsolateral (channels 41, 42, 44), and ventrolateral (channel 4) prefrontal regions. In summary, both VIIT and MICT effectively enhance executive function; VIIT offers a greater advantage in eliciting widespread prefrontal activation, whereas MICT demonstrates stable improvements in basic cognitive performance. These findings indicate that exercise can boost executive function and brain performance through neurophysiological mechanisms. Future studies could integrate psychology to explore how exercise interventions alleviate psychological stress and thereby enhance executive function and brain mechanisms.

## 6 Limitations

This study has several limitations in training-intensity design, the scope of cognitive-brain correlations, and sample representativeness. It did not systematically examine the dose–response relationship between different intensities and cognitive improvements, focusing only on the prefrontal cortex. The sample size was small (*n* = 17/group), exclusively male, and potential learning effects were not controlled. Additionally, differences in participants' prior exercise experience were not considered, which may affect the comparability of the results. Future research should expand the sample, include female participants, employ crossover or delayed post-test designs, integrate multi-region neuroimaging with graded intensity protocols, and account for individual exercise experience to further clarify the long-term cognitive benefits and neural mechanisms of exercise interventions.

## Data Availability

The original contributions presented in the study are included in the article/supplementary material, further inquiries can be directed to the corresponding author.
